# Supplementary material for: Terahertz and mid-infrared plasmons in three-dimensional nanoporous graphene
Source: Nat Commun. 2017 Mar 27;8:14885. doi: 10.1038/ncomms14885 (PMC5378955; doi:10.1038/ncomms14885)
Supplement: Supplementary Information — Supplementary Figures, Supplementary Notes, Supplementary Table and Supplementary References [file ncomms14885-s1.pdf]

Supplementary Note 1: Infrared Microscopy measurements

We performed infrared transmission measurements through an infrared microscope focusing the radiation over a 35 µm spot. The comparison among macroscopic IR data (2 mm spot) and microscopic data (35 µm spot) for different microscopic points inside the macroscopic spot and separated of several hundreds of microns is reported in Supplementary Figure 1b. This Figure shows very reproducible and substantial independent IR spectra from sample surface position (numbered by 1,2, 3 in Supplementary Figure 1a), at least down to micrometric spatial scale.

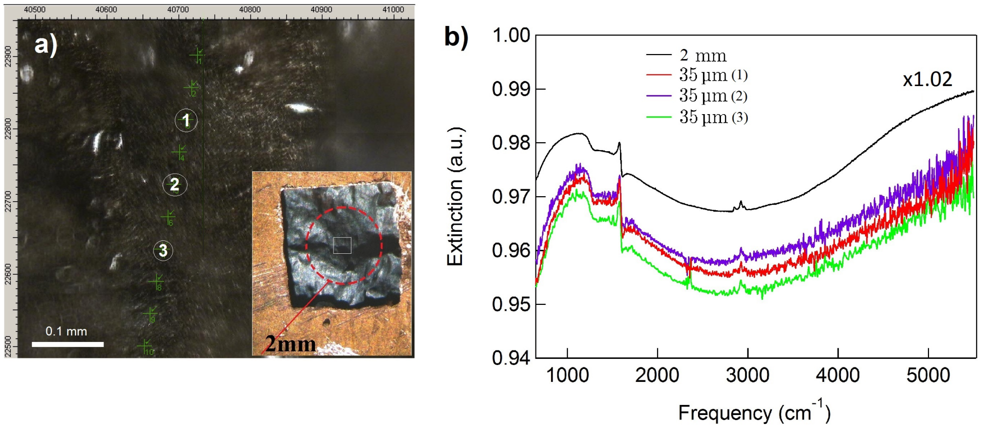

Supplementary Figure 1: **Comparison of spectra at different spatial resolution.** a) Microscopic image of NPG (inset shows the sample mounted free standing on its copper frame). b) absorption spectra for N-doped nanoporous graphene taken with an Infrared microscope (36x) and a spot size of 35 µm at different spacial location as indicated in the optical image a), to investigate inhomogeneity of the spectral response. For comparison the spectra acquired with the macroscopic FTIR beam (2 mm) is reported (black) for the same sample.

Supplementary Note 2: Optical Conductivity Model

The conductivity model used in this work is based on two components. The plasmonic peak is modelled with a Lorentz oscillator, its real part being [2]:

$$\sigma(\nu)_{plasm} = \frac{B\nu^2\Gamma_{pl}}{(\nu_{pl}^2 - \nu^2)^2 + (\Gamma_{pl}\nu)^2} \tag{1}$$

where B is the oscillator strength, ν_{pl} the plasmon frequency and Γ_{pl} its linewidth.
The second component takes into account the interband electronic transition in graphene [3, 4]:

$$\sigma_{inter}(\nu) = C\left[\tanh\left(\frac{h\nu - 2E_F}{4k_BT}\right) + \tanh\left(\frac{h\nu + 2E_F}{4k_BT}\right)\right] \tag{2}$$

where T is the temperature while C defines the intensity of the interband transitions, and depends on both the thickness and the effective number of graphene layers in the film.

A spatial inhomogeneity of the doping level and then of the Fermi Energy E_{F}, probably related to the nanostructuring of 3D graphene, is necessary to describe experimental data (see main manuscript). The Fermi energy inhomogeneity can be taken into account through a probability distribution $\mathrm{P}(E_{\mathrm{F}})$. An infrared spot size of a few millimeters in the far-field limit, corresponds mathematically to averaging Eq. 2 over $\mathrm{P}(E_{\mathrm{F}})$ through the equation

$$\sigma'_{inter}(\nu) = \int \sigma_{inter}(\nu, E'_F - E_F) P(E'_F) dE'_F. \quad (3)$$

An analytic expression of the previous integral can be obtained assuming for P(E_{F}) a flat distribution between two limiting values, E_{F_1} and E_{F_2}, which reads:

$$P(E_F) = \begin{cases} 0 & E_F < E_{F_1}; \quad E_F > E_{F_2} \\ \frac{1}{E_{F_2} - E_{F_1}} & E_{F_1} < E_F < E_{F_2} \end{cases} \quad (4)$$

The final expression of the optical conductivity, as analitically obtained from the previous Equation and then fitted to experimental data is:

$$\sigma(\nu) = A + \frac{B\nu^2\Gamma_{pl}}{(\nu_{pl}^2 - \nu^2)^2 + (\Gamma_{pl}\nu)^2} + C\frac{(k_BT)}{(E_{F_2} - E_{F_1})} \ln\left(\frac{\cosh\left(\frac{\nu+2E_{F_2}}{4k_BT}\right)\cosh\left(\frac{\nu-2E_{F_1}}{4k_BT}\right)}{\cosh\left(\frac{\nu-2E_{F_2}}{4k_BT}\right)\cosh\left(\frac{\nu+2E_{F_1}}{4k_BT}\right)}\right) \quad (5)$$

where A takes into account an absorption background also observed in single-layer graphene [5]. An example of fit through Eq. 5 is plotted in Fig. 1 of the main manuscript. Another example is reported on Supplementary Figure 2. In the same Figure we also plot a fit to the optical conductivity as obtained by a gaussian convolution model. In this case an analytic solution cannot be obtained and the calculation has been performed numerically through Mathematica.

Both fits well reproduce the experimental data providing consistent fit parameters, *i.e.* similar average Fermi energies, and plasmon characteristic frequencies and linewidths.

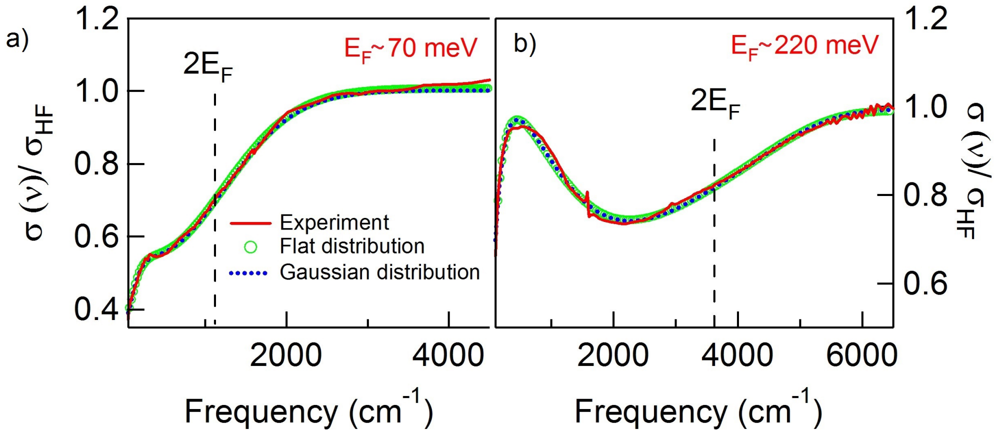

Supplementary Figure 2: **Conductivity models with different Energy distribution fucntions.** Experimental conductivity (red line) of undoped a) and Nitrogen-doped b) NPG samples. The conductivity analytical model well fits the experimental data assuming both a flat (green circles) or a gaussian distribution (blu dotted) of the Fermi energy values across the sample.

In Supplementary Table I, we report the result of fitting the optical conductivity model to the experimental data reported in Fig. 2 and 4 of the main manuscript. NP and NPN are relative to undoped and N-doped samples, respectively. The II, III, IV and Vth columns report the pore size p, the plasmon frequency ν_{pl} and widths Γ_{pl}, the average Fermi energy E_{F} and the corresponding statistical width. Let us note that data reported in Supplementary Table I correspond to an average on several samples having, nominally, the same physical properties.

Supplementary Note 3: Variation of optical properties

Several samples with nominally identical pore-size and doping were measured from the same batch obtaining consistent results (see Supplementary Figure 3). By taking into account both the interband threshold variation, and the fitting procedure (see Supplementary Equation 5), a relative uncertainty of about 10 % on the average Fermi energy is finally obtained. The relative uncertainty on the plasmon characteristic frequency is on the same order of magnitude.

Sample	p (nm)	$\nu_{\rm pl}$ (cm^{-1})	$\Gamma_{\rm pl}$ (cm^{-1})	$E_{\rm F}$ (cm^{-1})
NP1	200±50	280±30	440±45	570±150
NP2	650±90	150±15	480±50	560±300
NP3	900±100	60±5	190±20	580±260
NPN1	200±50	450±50	1550±150	1770±600
NPN2	200±50	670±70	2550±250	2100±600
NPN3	200±50	800±80	2450±250	2800±1000

Supplementary Table I: **Fitting parameters.** Values of fitting parameters for the conductivity spectra reported in the main manuscript.

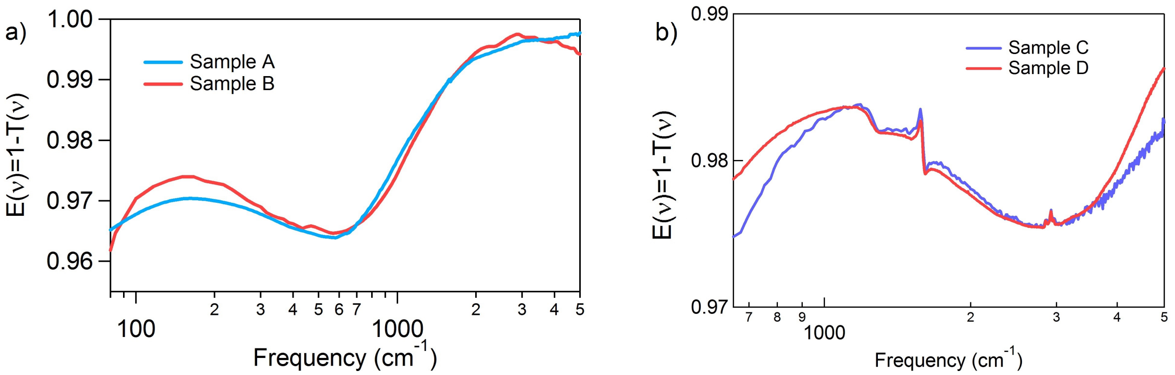

Supplementary Figure 3: **Spectra of samples from the same batch:** Extinction spectra of different samples from the same batch for undoped NPG a) and N-doped NPG b).

Supplementary Note 4: Distribution of plasmonic spectral weight.

While plasmons in 2D graphene exhibit an extinction peak from a few percent up to 25%, plasmonic excitation measured in 3D nanoporous graphene show an enhanced extinction, with values greater than 95% (see Fig.1 of the main manuscript). This enhancement is ascribable both to the large effective number of layers that constitute the 3D graphene configuration and on NPG microscopic polarizability. In order to quantitatively discuss the plasmon strength, we will compare the real part of the optical conductivity plasmons in both single-layer and 3D NPG systems. The strength of the plasmon peak in the optical conductivity (i.e. its spectral weight), is also a measure of the corresponding polarizabilities.

In Supplementary Figure 4 we show both the NPG plasmon band (for the NPG sample characterized by p=200 nm and $E_{\rm F}$=260 meV, see Fig 2g of the main manuscript), and for a single layer plasmon peak representative of data available in literature (corresponding to an extinction peak of 12%, a linewidth of Γ=120 cm^{-1}, and a central frequency of 750 cm^{-1}). The real part of the optical conductivity of these bands is normalized to their interband high frequency value $\sigma_{\rm HF}$.

The plasmon band in the optical conductivity provides a similar spectral weight (area under the plasmon peak) in both cases. This demonstrates that oscillator strengths of plasmons in NPG are comparable to that found in single-layer graphene although distributed over a larger spectral window due to geometrical and doping inhomogeneity. The broader footprint of NPG plasmons could be a useful feature when considering applications in Surface Enhanced Infrared Absorption.

Supplementary Note 5: Optical Isotropy

The isotropy of the optical response has been checked by measuring the extinction spectra in the mid-IR through a linear infrared polarizer at different polarizations. The data yield no appreciable variations (<1%) for two extreme angles, as reported in Supplementary Figure 5. This is in full agreement with photoemission measurements on samples of the same batch [1], which also show an isotropic response.

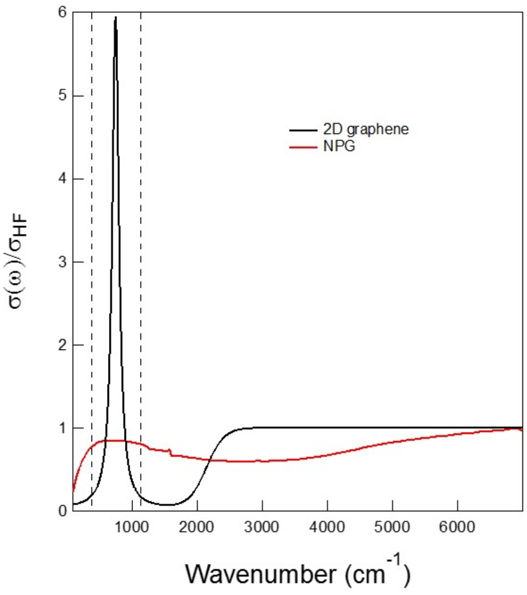

Supplementary Figure 4: **Plamsonic spectral weight for NPG and single-layer graphene model:** Infrared conductivity spectra of NPG (red) and monolayer graphene (black), normalized to the interband high-frequency value HF. Central frequency for both plasmons is around 800 cm-1. The black dashed vertical lines represents the minimum and maximum NPG plasmon frequency as due to statistical errors in both E_F and p.

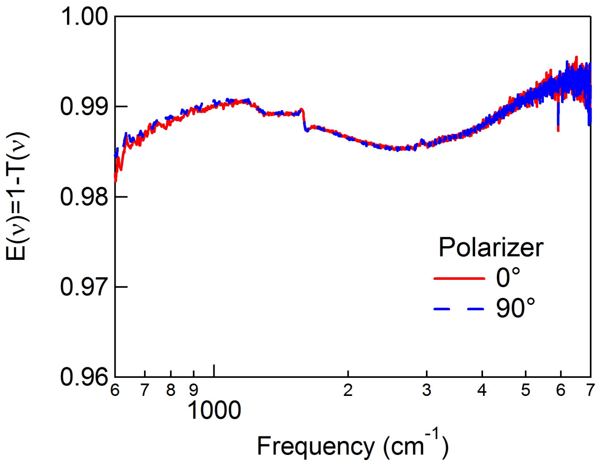

Supplementary Figure 5: **Light polarization dependence:** Extinction spectra for an N-NPG sample at orthogonal polarizations of the incident light.

Supplementary References

[1] Y. Ito, Y. Tanabe, H. J. Qiu, K. Sugawara, S. Heguri, N. H. Tu, K. K. Huynh, T. Fujita, T. Takahashi, K. Tanigaki, and M. Chen, High-quality three-dimensional Nanoporous Graphene, *Angew. Chem. Int. Ed.*, **126**, 1 (2014)

[2] M. Dressel, G. Gruner, Electrodynamics of Solids: Optical Properties of Electrons in Matter, *Cambridge* (2002)

[3] A. B. Kuzmenko, E. van Heumen, F. Carbone and D. van der Marel, Universal Optical Conductance of Graphite, *Phys. Rev. Lett.* **100**, 117401 (2008)

[4] K. F. Mak, L. Ju, F. Wang, T. F. Heinz, Optical Spectroscopy of Graphene: From the Far Infrared to the Ultraviolet, *Solid*

State Commun. **152**, 1341 (2012)

[5] Z. Q. Li, E. A. Henriksen, X. Jiang, X. Hao, M. C. Martin, P. Kim, H. L. Stormer and D. N. Basov, Dirac Charge Dynamics in Graphene by Infrared Spectroscopy, *Nat. Physics*, **4**, 532 (2008)
